# Fluorescence Characterization of Gold Modified Liposomes with Antisense *N-myc* DNA Bound to the Magnetisable Particles with Encapsulated Anticancer Drugs (Doxorubicin, Ellipticine and Etoposide)

**DOI:** 10.3390/s16030290

**Published:** 2016-02-25

**Authors:** Sylvie Skalickova, Lukas Nejdl, Jiri Kudr, Branislav Ruttkay-Nedecky, Ana Maria Jimenez Jimenez, Pavel Kopel, Monika Kremplova, Michal Masarik, Marie Stiborova, Tomas Eckschlager, Vojtech Adam, Rene Kizek

**Affiliations:** 1 Department of Chemistry and Biochemistry, Faculty of Agronomy, Mendel University in Brno, Zemedelska 1, CZ-613 00 Brno, Czech Republic; sylvie.skalickova@gmail.com (S.S.); lukasnejdl@gmail.com (L.N.); george.kudr@centrum.cz (J.K.); brano.ruttkay@seznam.cz (B.R.-N.); anuskajj@hotmail.com (A.M.J.J.); paulko@centrum.cz (P.K.); mkremplova@volny.cz (M.K.); michal.masarik@mail.muni.cz (M.M.); vojtech.adam@mendelu.cz (V.A.); 2 Central European Institute of Technology, Brno University of Technology, Technicka 3058/10, CZ-616 00 Brno, Czech Republic; 3 Department of Biochemistry, Faculty of Science, Charles University, Albertov 2030, CZ-12840 Prague, Czech Republic; marie.stiborova@natur.cuni.cz; 4 Department of Paediatric Haematology and Oncology, 2nd Faculty of Medicine, Charles University, and University Hospital, Motol V Uvalu 84, CZ-15006 Prague, Czech Republic; Tomas.Eckschlager@fnmotol.cz

**Keywords:** liposome, gold nanoparticles, *N-myc*, doxorubicin, ellipticine, etoposide

## Abstract

Liposome-based drug delivery systems hold great potential for cancer therapy. The aim of this study was to design a nanodevice for targeted anchoring of liposomes (with and without cholesterol) with encapsulated anticancer drugs and antisense *N-myc* gene oligonucleotide attached to its surface. To meet this main aim, liposomes with encapsulated doxorubicin, ellipticine and etoposide were prepared. They were further characterized by measuring their fluorescence intensity, whereas the encapsulation efficiency was estimated to be 16%. The hybridization process of individual oligonucleotides forming the nanoconstruct was investigated spectrophotometrically and electrochemically. The concentrations of ellipticine, doxorubicin and etoposide attached to the nanoconstruct in gold nanoparticle-modified liposomes were found to be 14, 5 and 2 µg·mL^−1^, respectively. The study succeeded in demonstrating that liposomes are suitable for the transport of anticancer drugs and the antisense oligonucleotide, which can block the expression of the *N-myc* gene.

## 1. Introduction

Liposomes are artificial spherical vesicles, which sizes range from tens of nanometres to several micrometres. They consist of one or more phospholipid bilayers surrounding an inner cavity. Synthetic or natural phospholipid molecules consist of polar head groups and hydrophobic hydrocarbon tails connected by a linker, such as glycerol [1]. Due to the fact that liposomes are primarily synthesized from biologically degradable materials, they are non-toxic and non-immunogenic. Due to their biocompatibility, size and hydrophobic and hydrophilic character, liposomes represent superb “micro-sized containers” suitable not only for delivery of drugs, but also for other functional biomolecules, such as nucleic acids and proteins [2,3,4,5]. Hydrophobic molecules can be loaded into the bilayer and hydrophilic ones are loaded into the cavity, where they are protected from degradation processes [6,7]. In addition, changes in the lipid composition, surface charge, and the method of preparation significantly affect their properties [1]. Due to this fact, liposomes have found application in medicine and pharmacy as delivery vehicles for drugs, antisense oligonucleotides, cloned genes, and recombinant proteins [8,9,10]. Moreover, it has been well documented that numerous anti-cancer agents were less-toxic in the liposomal form in comparison with conventional free drugs [11,12,13,14]. Besides delivery purposes, liposomes can be also used as a cell membrane model to study the function of membrane proteins in biophysics and in biochemistry [15,16].

Numerous scientists have been solving the issue how to deliver drugs selectively to the site of action. This issue is closely connected with lowering of doses, reducing drug waste, and eliminating the unwanted side effects of therapy. Nanogold-modified liposomes have found an application in many areas, especially in chemistry for free DNA sensing [17]. Moreover, gold has a great affinity to thiol groups and the self-assembly of an organic monolayer on gold is one of the best methods for surface modification of various materials. Some thiol-containing compounds can be easily removed from the Au surface, thus, they have found applications where temporary nanoscale coatings were required [18]. Utilization the gold nanoparticles (AuNPs) modified liposome as a nanotransporter device with attraction to thiol modified oligonucleotides has been published, too [19].

The aim of this study was to suggest and develop a modular nanodevice consisting of AuNPs-modified liposomes with enclosed anticancer drugs and the antisense oligonucleotide, which can block the expression of the selected gene. Doxorubicin, ellipticine and/or etoposide were used as examples (due to their fluorescent properties), however, any other drug may be used. *N-myc* gene was used as a model antisense oligonucleotide to demonstrate the concept.

## 2. Experimental Section

### 2.1. Chemicals and Material

Anticancer drugs (doxorubicin, ellipticine and etoposide), cholesterol, 1,2-dioleoyl-*sn*-glycero-3-phospho-*rac*-(1-glycerol) sodium salt, chloroform, sodium citrate, HAuCl_4_·3H_2_O and other reagents were purchased from Sigma-Aldrich (St. Louis, MO, USA) in ACS purity (*i.e.*, purity meeting the standards of the American Chemical Society), unless noted otherwise. Hydrogenated phosphatidylcholine from soybean was a gift from Lipoid GmbH (Ludwigshafen, Germany). Magnetic particles (MPs) oligo(DT)_25_ were purchased from Invitrogen (Oslo, Norway). The deionised water was prepared using Aqual 25 reverse osmosis equipment (Aqual, Brno, Czech Republic). The deionised water was further purified by using a MilliQ Direct QUV apparatus equipped with an UV lamp. The resistance was 18 MΩ. The pH was measured using a WTW inoLab pH meter (WTW GmbH, Weilheim, Germany).

### 2.2. Preparation of Gold Nanoparticles (AuNPs)

Gold nanoparticles were prepared by citrate method at room temperature according to Kimmling *et al.* and Polte *et al.* [20,21]. Briefly, an aqueous solution of sodium citrate (0.5 mL, 40 mM) was added to a solution of HAuCl_4_·3H_2_O (10 mL, 1 mM). The colour of the solution slowly changed from yellow to violet. The mixture was stirred overnight.

### 2.3. Preparation of Liposome Film and Liposome Encapsulating Doxorubicin, Ellipticine and/or Etoposide

Liposomes were prepared according to a published method [22] with some modifications. Briefly, cholesterol (100 mg), 1,2-dioleoyl-*sn*-glycero-3-phospho-*rac*-(1-glycerol) sodium salt (100 mg) and phosphatidylcholine (100 mg) were dissolved in chloroform (4.5 mL). Cholesterol-free liposomes were prepared in the same manner and dissolved in 3.5 mL of chloroform. A lipid film was obtained by rotary evaporation of chloroform. Residual chloroform was removed by a stream of nitrogen. Solutions containing 500 µg of the anticancer drugs and water (500 µL) were added to the lipid film (10 mg). Samples were homogenized with an Ultra-Turrax T8 (IKA Werke GmbH, Staufen, Germany) for 10 min. The homogenized mixtures were then heated and shaken for 15 min at 60 °C at a Thermomixer Comfort (Eppendorf, Hamburg, Germany). The samples were then washed several times with MiliQ water on an Amicon 3k (Merck Millipore, Merck KgaA, Darmstadt, Germany). The final volume of samples was 1 mL.

### 2.4. Modification of Liposome Surface by AuNPs

Modification of liposomes was done according to Bhuvana *et al.* [17]. Liposomes containing anticancer drugs prepared as described in the previous section were used. After cooling, a 1 mM solution (500 µL) of gold nanoparticles was added. The mixture was shaken for 3 h on a Biosan Orbital Shaker OS-10 (Biosan Ltd., Riga, Latvia). Subsequently, the volume was filtered through Amicon 3K (Merck Millipore, Merck KgaA, Darmstadt, Germany) under the following conditions: 3500 rpm, 20 °C, and 15 min. The mixture was washed several times with MilliQ water and finally diluted to 1 mL.

### 2.5. Amplification of Exon 2 of Human N-myc Gene

Neuroblastoma cells were obtained from the Faculty of Science, Masaryk University (Brno, Czech Republic). Isolation of genomic DNA of neuroblastoma cells were performed using a MagNA Pure Compact, Nucleic Acid Isolation Kit I (Roche, Mannheim, Germany), according to the manufacturer’s instructions. The sequence was obtained from the GenBank database; with the following accession number X03294.1. One part of the exon 2 of human *N-myc* gene was obtained by polymerase chain reaction (PCR) amplification of genomic DNA, using a set of primers (5-ATGCCGGGCATGATCTGC (h-*N-myc*-exon2-fw) and 5-TGCAGCTTCTCGCTCA (h-*N-myc*-exon2-rev)). The PCR products were purified using the MinElute PCR Purification Kit (Quiagen, Hilden, Germany).

### 2.6. Isolation of AuNPs-Modified Liposomes Using Magnetic Microparticles

For isolation of nanogold-modified liposomes, magnetic particles modified by polythymine chain (25 thymines) (Invitrogen, Oslo, Norway) were used. The solution used for washing MPs contained 0.1 M NaCl and 0.05 M phosphate buffer. The hybridization solution was as follows: 0.1 M Na_2_HPO_4_; 0.1 M NaH_2_PO_4_; 0.6 M guanidinium thiocyanate (Amresco, Solon, OH, USA); 0.15 M Tris-HCl (pH 7.5); and 0.2 M NaCl. For the first hybridization 10 µL of MPswas pipetted to an Eppendorf tube placed in a magnetic holder and washed three times with 100 µL of the phosphate buffer. Afterwards, 10 µL of oligodeoxynucleotide (100 µM) 5′ AAAA AAAA AAGA CTGG GTAG TTAA CCTT ACGT CT-SH 3′ and 10 µL of hybridization buffer was added and incubated (30 min, 25 °C) on a Multi RS-60 (Biosan Ltd.). Sample was washed three times with 100 µL of phosphate buffer using the magnetic holder. Subsequently, the elongated antisense *N-myc* oligonucleotide (*as-N*-*myc*) (5′-GTTC-TTGC-AGAT-CATG-CCCG-CTGA-CCCA-TCAA-3′) was attached to magnetic particle oligonucleotide probe (MPs-ODN probe). To 10 µL of MPs-ODN probe in the phosphate buffer 10 µL of *as-N-myc* ODN (100 µM) was pipetted and washed three times with 100 µL of phosphate buffer. Subsequently, the *N-myc* gene from exon 2 was attached in the same way. The 10 µL of *N-myc* gene sequence (100 µM) was incubated (30 min, and 25 °C) on Multi RS-60 (Biosan Ltd.) and afterwards washed three times with 100 µL of the phosphate buffer. The last step was labelling of 10 µL of nanoconstruct by 10 µL of anticancer drugs encapsulated into AuNPs modified liposome. The mixture was incubated (30 min, ad 25 °C) and washed using the magnetic holder three times with the phosphate buffer (3 × 100 µL). Afterwards, 10 µL of water in ACS purity was added and incubated (5 min, 95 °C, and 14,000 rpm) on a Thermomixer Comfort (Eppendorf). Finally, samples were rapidly cooled on ice. Magnetic particles were separated by a magnet and the solution was used for following measurements. Other experimental details can be found in [23,24,25,26,27].

### 2.7. UV/VIS Spectroscopy

Fluorescence spectra were acquired by a Tecan Infinite 200 PRO multifunctional microplate reader (TECAN, Mannedorf, Switzerland). Excitation wavelength for ellipticine, doxorubicin and etoposide was 420, 480 and 250 nm, respectively. The fluorescence scan of ellipticine, doxorubicin and etoposide was measured within the range from 450 to 850 nm, 510–850 nm and 280–850 nm, respectively, per 2-nm steps. The detector gain was set to 100. Absorbance of ssDNA was measured at λ = 260 and 280 nm. Each absorbance value is an average of three measurements. The samples for both measurements (2 µL) were placed in a 16 well Tecan NanoQuant plate. All measurements were performed at 30 °C controlled by the Tecan Infinite 200 PRO.

### 2.8. Determination of ODN-CA Peak

Determinations of ODN by square wave voltammetry were performed by a 663 VA Stand instrument (Metrohm, Herisau, Switzerland) connected with an AUTOLAB Analyzer (Metrohm) using the adsorptive transfer technique and a standard cell with three electrodes. A hanging mercury drop electrode (HMDE) with a drop area of 0.4 mm^2^ was the working electrode. An Ag/AgCl/3M KCl electrode was the reference and platinum electrode was auxiliary. For data processing GPES 4.9 software by Metrohm was employed. The analysed samples were deoxygenated prior to measurements by purging with argon (99.999%). For CA peak analysis the Tris buffer (10 mM Trizma base adjusted to pH = 5 with acetic acid) was used as a supporting electrolyte. The parameters of the measurement were as it follows: initial potential 0 V, end potential −1.7 V, deoxygenating with argon 30 s, accumulation time 120 s, step potential 5 mV, modulation amplitude 25 mV, volume of injected sample: 5 µL, and volume of measurement cell: 2 mL (5 μL of sample and 1995 µL supporting electrolyte).

### 2.9. Descriptive Statistics

Data were processed using EXCEL^®^ (Microsoft, Redmond, WA, USA). Results are expressed as mean ± standard deviation unless noted otherwise (EXCEL^®^).

## 3. Results and Discussion

In this study, we focused on the preparation of a modular nanodevice combining the transport of an anticancer drug and silencing oligonucleotide able to block the selected gene. To prove this concept, three fluorescent anticancer drugs (doxorubicin, etoposide and ellipticine) as well as *N-myc* gene were used, however due to the modularity of the construct any other drug/gene may be used. To effectively couple the components and remove the unreacted molecules, the benefits of attachment to magnetic particles were exploited. The manipulation of the nanoconstruct by the external magnetic field simplifies the preparation and purification.

### 3.1. Characterization of Fluorescence Behaviour of Enclosed Drugs in Liposomes

**Figure 1:**
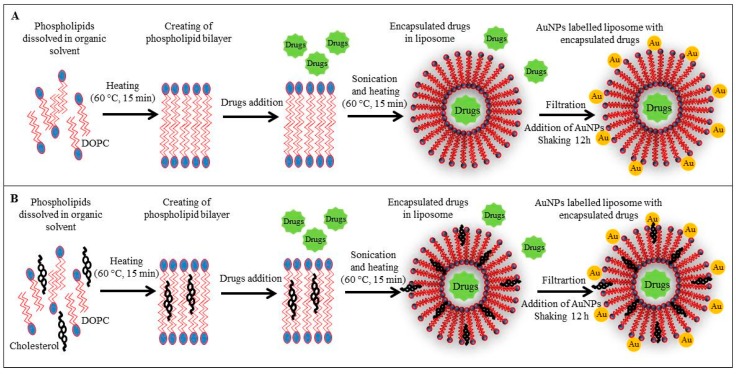
The experimental scheme of encapsulation of drugs in AuNPs-labelled liposomes. (**A**) Phosphatidylcholine liposome; (**B**) Phosphatidylcholine-cholesterol liposome. Other experimental conditions is in Experimental Section.

The first step consisted of enclosing of the anticancer drugs in liposomes with subsequent modification of their surface with AuNPs. First, the liposome without cholesterol (LIP-10, Figure 1A) and cholesterol-containing liposome (LIP-8, Figure 1B) were used for encapsulation of the anticancer drugs doxorubicin, ellipticine or etoposide exhibiting fluorescence properties. Both liposome variants were modified by AuNPs for specific bonding to thiolated oligonucleotide used as a carrier of antisense *N-myc* gene.

We used fluorescence spectroscopy for analysis of the intensity of doxorubicin, etoposide and ellipticine fluorescence. Firstly, we determined the dependence of fluorescence intensity on drug concentration.

**Figure 2:**
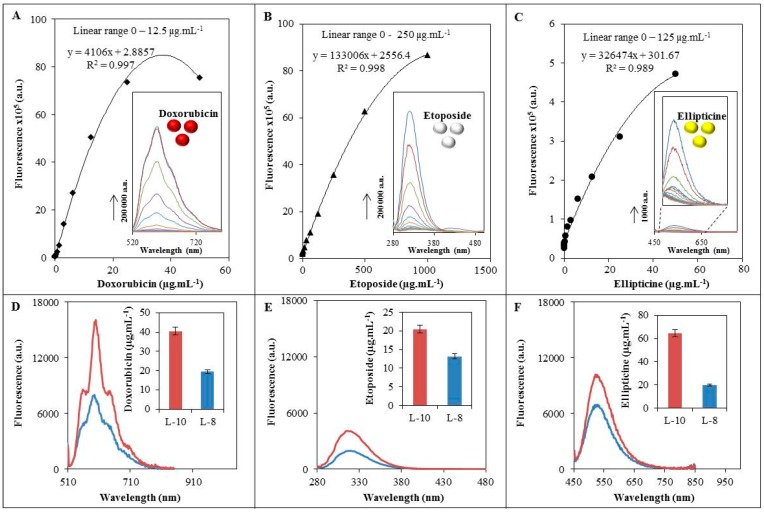
Dependence of fluorescence signal on drug concentration. The corresponding fluorescence spectra are given in the insets. (**A**) Doxorubicin within the 0.05–50 µg·mL^−1^ concentration range; (**B**) etoposide within the 1–1000 µg·mL^−1^ concentration range and (**C**) ellipticine within the 0.05–50 µg·mL^−1^ concentration range. Fluorescence spectra of encapsulated drugs in cholesterol-free liposomes (red line) and liposomes with cholesterol (blue line): (**D**) doxorubicin; (**E**) etoposide; and (**F**) ellipticine. The calculated encapsulated concentration of drug in liposomes is given in the inserted graphs. Excitation wavelengths for doxorubicin, etoposide and ellipticine were 480, 250 and 420 nm, respectively.

The excitation wavelengths of doxorubicin, etoposide and ellipticine were *λ* = 480, 250 and 420 nm, respectively, and emission was recorded within the ranges of *λ* = 510–850, 280–850 and 450–850 nm, respectively. In the case of doxorubicin, the calibration curve within the range of 0.05–50 µg·mL^−1^ showed a polynomial trend fitting the following equation in the linear part (0.05–12.5 µg·mL^−1^): *y* = 4106*x* + 2.8857 and *R*^2^ = 0.997 (Figure 2A). For etoposide, a polynomial calibration curve within the range of 1–1000 µg·mL^−1^ was obtained. The linear part within the range from 1 to 250 µg·mL^−1^ fitted the following equation *y* = 133006*x* + 2556.4 and *R*^2^ = 0.998 was determined (Figure 2B). Finally, the dependence on the ellipticine concentration within the 0.05–50 µg·mL^−1^ range showed a polynomial trend and the regression equation of the linear part in the 0.05–30 µg·mL^−1^ range *y* = 326474*x* + 301.67 and *R*^2^ = 0.9893 was obtained, see Figure 2C. The observed polynomial trend of the calibration curve in all fluorescence label cases is caused by quenching of the fluorescence due to the high concentration [28,29]. To ensure that the fluorescence quenching did not occur in the linear part of the calibration curve, we diluted the anticancer drugs accordingly for further experiments.

Drug loading and encapsulation efficiency is very important in a drug delivery system [30]. We evaluated the encapsulation efficiency of the studied anticancer drugs in the liposomes prepared with cholesterol (LIP-8) and cholesterol free liposomes (LIP-10) by comparison of the anticancer drugs fluorescence encapsulated and non-encapsulated in the liposome before and after addition of 3% sodium dodecyl sulphate (SDS), which caused the drug release from the liposomes. Firstly, samples were diluted to the concentration in the linear range of each anticancer drug to avoid any fluorescence quenching. The concentration of each drug encapsulated into the liposome was estimated to be 160 µg·mL^−1^ which is 16% of the applied concentration of 1000 µg·mL^−1^ as described in the Experimental Section (data not shown). Cholesterol is often included in liposome formulations to give further rigidity to the bilayer that may improve the *in vivo* and *in vitro* stability of the liposomes [31]. We studied the fluorescence behaviour of liposomes with cholesterol and cholesterol-free liposomes. The fluorescence spectra in Figure 2D–F show different fluorescence intensities of anticancer drugs for both varieties of liposomes. The intensity of emission of the doxorubicin encapsulated in LIP-8 or LIP-10 at *λ* = 510 nm was 16,000 arbitrary units (a.u.) and 8000 a.u., respectively. The differences in emissions between LIP-8 and LIP-10 corresponded to 50% of LIP-10 (Figure 2D). From Figure 2E it is evident that the intensity of emission of the etoposide encapsulated in LIP-8 or LIP-10 at *λ* = 250 nm was 4000 a.u. or 1900 a.u., respectively. The difference in emissions corresponded to 47% of LIP-10. Finally, the intensity of emission of the ellipticine encapsulated in LIP-8 or LIP-10 at *λ* = 510 nm was 10,000 a.u. or 7000 a.u., respectively. The difference in emissions corresponded to 68% of LIP-10. In the case of the unmodified liposomes and liposomes modified with AuNPs, no change in emission (fluorescence) was observed (data not shown). The differences in fluorescence between both types of liposomes are probably caused by cholesterol addition, which is in good agreement with previously reported data [32]. Quenching of fluorescence by cholesterol was also described e.g., for human serum albumin [33].

### 3.2. Hybridization of ODN

**Figure 3:**
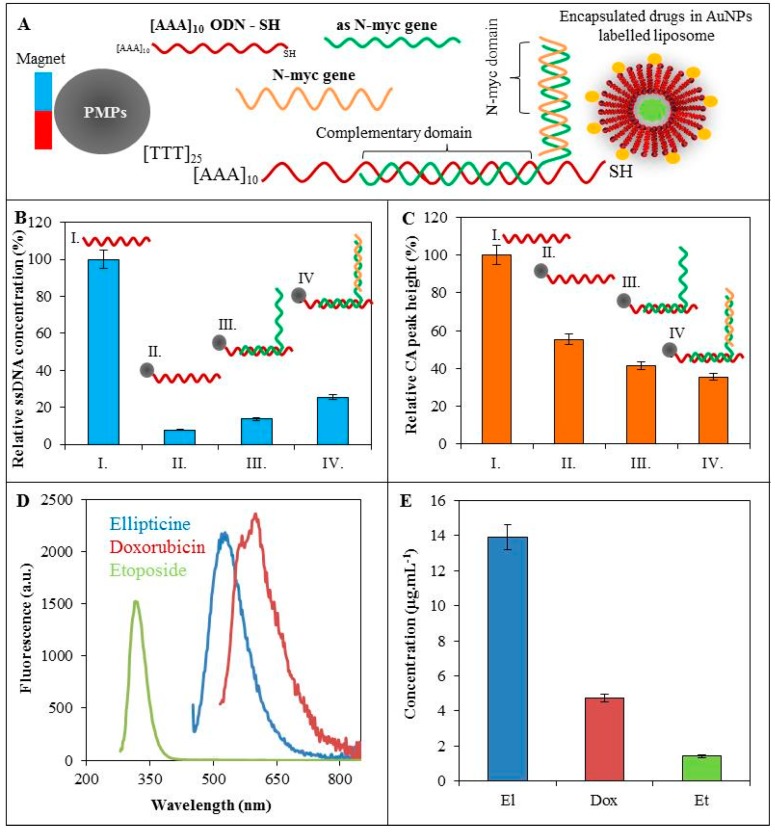
(**A**) Scheme of the nanoconstruct of the thymine modified paramagnetic particles (PMPs), which were bound with (AAA)_10_ODN-SH based on their complementarity. On the thiolated ends of oligonucleotides the AuNPs were tied to modified liposome with encapsulated fluorescence label. On the complementary domain of (AAA)_10_ODN-SH the *as-N-myc* sequence was hybridized for binding to *N-myc* gene; (**B**) Spectrophotometric analysis of relative ssDNA concentration and (**C**) electrochemical analysis (square wave voltammetry) of relative CA peak height during hybridization of: (I.) 50 µg·mL^−1^ (AAA)_10_ODN-SH isolated by (II.) thymine labelled MPs and (III.) attached 50 µg·mL^−1^ elongated *as-N-myc* sequence for (IV) binding of 50 µg·mL^−1^ *N-myc* gene. During the individual hybridization steps, samples were washed with phosphate buffer; (**D**) Fluorescence spectra of isolated drugs (ellipticine, doxorubicin, or etoposide, all in concentrations of 160 µg·mL^−1^). Excitation wavelengths for ellipticine, doxorubicin and etoposide were 420, 480 and 250 nm, respectively; (**E**) Concentration yield of encapsulated ellipticine, doxorubicin, or etoposide (160 µg·mL^−1^) in the liposome after nanoconstruct isolation.

MPs are a suitable tool for DNA isolation and manipulation allowing for the purification of DNA from various matrices [34,35,36,37]. Various modifications of the magnetic microparticles could provide the specific binding to analytes [38,39,40]. In our case, we used thymine-labelled magnetic particles for coupling of the (AAA)_10_ODN-SH. To this sequence, the oligonucleotide (*as*-*N-myc* gene) was hybridized. ODN sequence is specially designed to bind partially to (AAA)_10_ODN-SH and partially to *N-myc* gene. The scheme of the nanoconstruct is shown in Figure 3A. To be effective, a high oligonucleotide hybridization yield is required. Hybridization yield depends on numerous factors, including nucleotide sequence, temperature, salt concentration and the time of hybridization. Based on our previously published results, we estimated the optimal conditions for hybridization process [23,24,25,26,27], which was confirmed spectrophotometrically and electrochemically. Primarily, we investigated the relative ssDNA concentration using spectrophotometric analysis (Figure 3B). The 100% is expressed as 50 µg/mL (AAA)_10_ODN-SH concentration before isolation by magnetic particles (I, Figure 3B). Afterwards, the 90% decrease of relative ssDNA concentration occurred after the isolation of the oligonucleotide (II, Figure 3B). The isolated ODN concentration is limited by binding capacity of magnetic particles. Binding the *as-N-myc* sequence (III, Figure 3B) caused a relative concentration increase to 13% and finally, the nanoconstruct was tested to be capable of binding of *N-myc* gene (50 µg/mL), which caused a 26% relative concentration increase.

Subsequently, electrochemical analysis of hybridization process was performed (Figure 3C). The nucleic acids have been reported to give redox common signals of adenine and cytosine (CA peak) at −1.4 V potential. It is commonly known that the ssDNA provides a higher electrochemical signal in comparison with dsDNA on HMDE. The highest signal of CA peak was found for (AAA)_10_ODN-SH (I). After the isolation of the ODN by paramagnetic particles (II), the relative CA peak height decreased to 55%. The attachment of *as-N-myc* sequence (III) caused the relative peak height decrease to 41%. Relative CA peak height of nanoconstruct with bonded *N-myc* gene was estimated to 35%.

### 3.3. Labelling of ODN

Due to the high affinity of gold for thiol groups (-SH), (AAA)_10_ODN-SH was attached to AuNPs-modified liposome with various encapsulated anticancer drugs, which were characterized in Section 3.1. Fluorescent drugs such as doxorubicin are suitable tools for *in vivo* imaging of cell systems [41]. Since the fluorescent labels encapsulated into LIP-8 showed a lower fluorescence we decided to use the LIP-10 variant for further experiments. Using the protocol mentioned above we built a nanoconstruct with the attached fluorescence label, which was purified using paramagnetic particles. Due to the limited binding capacity of MPs, only a fraction of the total liposomal drug (160 µg·mL^−1^) is bound to the MPs and is able to go through the whole magnetic field isolation process. It clearly follows from the results obtained and shown in Figure 3C that the emission intensity of the doxorubicin encapsulated in LIP-10 at λ = 600 nm corresponded to an isolated doxorubicin concentration of 5 µg·mL^−1^. The ellipticine emission intensity corresponded to an isolated concentration ellipticine of 14 µg·mL^−1^. Finally, the emission intensity of etoposide at λ = 320 nm corresponded to an isolated etoposide concentration of 2 µg·mL^−1^.

## 4. Conclusions

In this paper, we presented a nanoconstruct which carries the *as-N-myc* gene and a fluorescent label formed by anticancer drugs with fluorescence properties (doxorubicin, ellipticine, or etoposide) encapsulated into the liposome modified by AuNPs. Due to its specific structure, this construct has the ability to turn off the *N-myc* gene, which promotes the progression of neuroblastoma and the presence of fluorescent drugs encapsulated into liposomes enables *in vivo* imaging of nanoconstruct transport into the target cell. Connectivity of nanoconstruct is mediated by repeating adenine sequence, which is complementary to thymine labelled magnetic particle and enables the purification of resulting nanoconstruct. Our results confirmed the decrease of the fluorescence of doxorubicin, ellipticine and etoposide by cholesterol, which naturally occurs in biological lipid membranes of eukaryotic cells. The concentrations of doxorubicin, ellipticine, and etoposide were found to be in the range of 5, 2 and 14 µg·mL^−1^ respectively, which is sufficient for *in vivo* imaging of treated cancer cells. Because of its properties, our construct should be a useful tool for gene therapy as well as for cell labelling.
